# Journal of Taibah University Medical Sciences: 16 years of success, and the journey continues

**DOI:** 10.1016/j.jtumed.2022.01.001

**Published:** 2022-01-17

**Authors:** Abdulmohsen Al-Zalabani, Fawzia Habib

**Affiliations:** a Department of Family and Community Medicine, College of Medicine, Taibah University, Al-Madinah, Saudi Arabia; b Department of Obstetrics and Gynecology, College of Medicine, Taibah University, Al-Madinah, Saudi Arabia

In December 2006, the first issue of the Journal of Taibah University Medical Sciences (JTUMS) was released.^1^ JTUMS started as in-house publication in Taibah University and then in 2012 it partnered with Elsevier to be the publisher of JTUMS. This year, the journal completed an exciting journey of 16 years. During this period, 16 volumes and 60 issues were published. The frequency of the journal publication increased from 2 issues annually to 6 issues per year accompanied by a constant increase in the number of submissions and published articles. Nonetheless, it was not only the quantity but also the quality of articles that was reflected in the increased impact and reach of the journal. Authors who published in JTUMS in the last 5 years came from over 30 countries in various continents. Reviewers were even more diverse and extended over the whole world. JTUMS has been indexed in SCOPUS, PubMed Central, and lately Emerging Sources Citation Index (ESCI).^2^ Each indexation was based on several quality criteria that were successfully satisfied by JTUMS. For example, the inclusion in ESCI requires journals to pass 24 quality criteria assessing editorial rigor and best practice in scholarly publishing.^3^ In addition, CiteScore of the journal increased from 0.9 in 2016 to 1.7 in 2020 as one indication of higher reach and impact of JTUMS.^4^ The Source-Normalized Impact per Paper (SNIP), which measures the citation impact of papers in a journal weighted by the total number of citations in a subject field, has doubled in the same period (from 0.542 in 2016 to 1.052 in 2020). All these milestones would not be possible without the hard work, support, and contribution of authors, reviewers, editors, and publication team as well as the support of Taibah University administration.

At this stage, Prof. Khalid Khoshhal (former Editor in chief) and Prof. Salman Guraya (former Deputy Editor in chief) decided to leave the journal to pursue new endeavors. We are deeply thankful to them for what they achieved in JTUMS and wishes them a fruitful time ahead. A new team of editors from various specialties has been appointed. The team, supported by a diverse editorial board spanning five countries, aims to take the journal to the next levels. To achieve this aim, five goals have been defined: 1) provide an added value to the readers; 2) expand and diversify authorship and readership of the journal; 3) build capacity of high-quality peer-review in the region; 4) build capacity of scholarly publication and editorial leadership in the region; and 5) help advance the culture of research quality, standards, ethics, and integrity. Several initiatives are underway to help achieve each goal and will be announced over the next few months.

We are extremely excited to start this journey and we want to thank the Scientific Council for electing us to lead the journal in the coming term. Special thanks go to our submitting authors who selected JTUMS to publish the product of their hard work, and our reviewers who dedicated their time and effort to promote science and support authors in publishing high quality articles.
